# To Properly Articulate a Set of Teeth

**Published:** 1894-07

**Authors:** J. G. Templeton


ARTICLE III.

### TO PROPERLY ARTICULATE A SET OF TEETH.

Having to make a full upper set of teeth, we will suppose the impression and model to have been made in the usual way. Take modeling composition, soften and flatten it out till it is about a quarter of an inch thick; press it on the model while warm, and then cut and trim to make a trial plate for the purpose of taking a bite. It should accurately fit the model. Melt a little wax around on the ridge then press a roll of softened wax on that, and trim to what you think would be a sufficient length, then try in the mouth and carefully trim the lower edge to the proper length for the teeth; if it is not, either add to or cut away till it is found by trying in the mouth that the wax represents the proper length. This wax should be so cut on its articulating surface that all the lower natural teeth will strike it at the same time when tried in the mouth. Now remove and soften the articulating wax surface just a little over the flame, then replace in the mouth, and do not let patient bite into it till you have the head drawn well back so as to put the anterior muscles of the neck on a stretch; then have the patient bite a little on the wax just to get an impression of the cusps and cutting edges of all the lower teeth. Next take an accuratc impression of the lower teeth, from which make a plaster model, which will fit into the slight impression of the teeth made in the bite taken, and then place the whole on any good articulator which can be set to maintain the relative positions. Remove the bite, and you are ready to set the teeth to a correct articulation, and if all has been carefully done the teeth will come together properly without any subsequent grinding.

For a double set (upper and lower) make trial plates of modeling composition to take the bite on, putting a piece of rather stiff wire in the lower one to stiffen it. Wax the ridges as previously described. Place a roll of softened wax on the upper trial plate, place the lower trial plate in the mouth, being careful to see that it is in its proper place, and hold it there while putting in the upper plate with the wax on it. Do not allow the patient to bite till the head is drawn back as far as you can get it; then tell the patient to bite, and then keep the jaws closed till with one finger the wax has been well pressed on the trial plate. Mark the center or medium line on the wax. Have patient close the lips, and then take a small, straight instrument and mark on the wax the height of the lower lip. This mark should extend from one angle of the mouth to the other; you then have the line of fissure or line of lip-closure; in other words, the height of the lower lip and length of he upper to serve as a guide in making the wax models. After thus taking the bite, place each of the models in the bite so obtained, and fasten in any good articulator; then prepare corresponding wax models, which should be tried in the mouth to verify their correctness. They should come together in the mouth the same as on the articulator and if they do not they should be made to do so before proceeding further. Take pains to be satisfied that the wax models are correctly adjusted and give a natural expression to all the facial features, observing that the lower third of the wax models is in proper proportion or length with the upper two-thirds, and be sure to produce the proper fullness over the region of the upper cuspids to give as near as possible the natural contour. Then take the upper and lower plaster models off the metal articulator, and make a plaster extension to the back part of upper model, on which place the wax models, which have been marked while in the mouth so that they can be put in the same position out of the mouth. The lower plaster model is placed in position, and a plaster extension added to fit to that of the upper plaster model. After separating these, the lower wax model is placed on the lower plaster model, and the inside space filled with wet paper, and plaster is poured over all to make the lower articulating plate to which the lower teeth are to be set. Next place the upper model in position, and set the upper teeth to the lower ones which have just been set to the lower articulating plate, and when ready for flasking, if for vulcanite plates, saw off articulating ends. Always set the lower teeth first.

Having made double sets in this way for twenty-five years without having to do any grinding after placing them in the mouth, I think that I have some claim to the conclusion that this method is a pretty good one.—*J. G. Templeton, in Cosmos.*

